# In vivo interaction of anti-cancer drugs with misonidazole or metronidazole: cyclophosphamide and BCNU.

**DOI:** 10.1038/bjc.1980.335

**Published:** 1980-12

**Authors:** I. F. Tannock

## Abstract

The addition of misonidazole (MISO) or metronidazole (METRO) to treatment with cyclophosphamide (CY) increased delay to regrowth of 2 experimental tumours. The effect was observed for large an small tumours, was present for doses of MISO that are ineffective for killing hypoxic cells, and required that it be given with, or shortly before CY. Mice receiving combined treatment had more weight loss and myelosuppression than those receiving CY alone, and the Therapeutic Index was lower. MISO caused a marked increase in growth delay when combined with BCNU to treat the KHT sarcoma. This effect was observed for small and large tumours, required simultaneous administration of drugs, and also led to increased host toxicity. There was no therapeutic advantage from combined treatment. Survival of aerobic or anoxic Chinese hamster ovary (CHO) cells was assessed after exposure in vitro to serum from mice that had received CY or BCNU alone. MISO alone, or combined treatment. Results of these experiments suggest that (1) MISO delays the excretion or breakdown of active metabolites of CY, and (2) at a dose that does not kill hypoxic cells, it may selectively "sensitize" hypoxic cells (but not aerobic cells) to the action of BCNU. The presence of other undetermined interactions of BCNU and MISO is inferred from the increased toxicity to (aerobic) normal tissue. Misonidazole or metronidazole should be used with caution in patients who are receiving BCNU or cyclophosphamide.


Br. J. Cancer (1980) 42, 871

### *IN VIVO* INTERACTION OF ANTI-CANCER DRUGS WITH MISONIDAZOLE OR METRONIDAZOLE: CYCLOPHOSPHAMIDE AND BCNU

I. F. TANNOCK

*From the Departments of Medicine and Physics, Ontario Cancer Institute and The Princess Margaret Hospital, Toronto, Canada M4X 1K9*

Received 24 June 1980 Accepted 26 August 1980

**Summary.—The addition of misonidazole (MISO) or metronidazole (METRO) to treatment with cyclophosphamide (CY) increased delay to regrowth of 2 experimental tumours. The effect was observed for large and small tumours, was present for doses of MISO that are ineffective for killing hypoxic cells, and required that it be given with, or shortly before CY. Mice receiving combined treatment had more weight loss and myelosuppression than those receiving CY alone, and the Therapeutic Index was lower.**

**MISO caused a marked increase in growth delay when combined with BCNU to treat the KHT sarcoma. This effect was observed for small and large tumours, required simultaneous administration of drugs, and also led to increased host toxicity. There was no therapeutic advantage from combined treatment.**

**Survival of aerobic or anoxic Chinese hamster ovary (CHO) cells was assessed after exposure *in vitro* to serum from mice that had received CY or BCNU alone, MISO alone, or combined treatment. Results of these experiments suggest that (1) MISO delays the excretion or breakdown of active metabolites of CY, and (2) at a dose that does not kill hypoxic cells, it may selectively "sensitize" hypoxic cells (but not aerobic cells) to the action of BCNU. The presence of other undetermined interactions of BCNU and MISO is inferred from the increased toxicity to (aerobic) normal tissue.**

**Misonidazole or metronidazole should be used with caution in patients who are receiving BCNU or cyclophosphamide.**

CYCLOPHOSPHAMIDE (CY) was reported to kill selectively the well-oxygenated cells of a transplantable rat tumour, and to spare the hypoxic, radioresistant cells (Dixon *et al.*, 1978). A similar effect was reported for the action of BCNU against B16 melanoma in mice, but CY had no selectivity for well-oxygenated cells of this tumour (Hill & Stanley, 1975). The mechanism of these effects is unknown, but potential causes of drug resistance of hypoxic and poorly nourished cells in solid tumours include limited diffusion of the drugs or their active metabolites from blood vessels, and a lower rate of cell proliferation for poorly nourished cells than for those situated closer to blood vessels (Tannock, 1968, 1970; Hirst & Denekamp, 1979).

If hypoxic cells in some solid tumours are resistant to CY or BCNU, an improved therapeutic index (*i.e.* ratio of tumour damage to normal tissue damage) might be achieved by combining them with agents that have selective toxicity for hypoxic cells. MISO and METRO are drugs that are known to have selective toxicity for hypoxic cells in tissue culture and in spheroids (Mohindra & Rauth, 1976; Sridhar *et al.*, 1976; Stratford & Adams, 1977; Taylor & Rauth, 1978) and have been shown to kill hypoxic (and perhaps neighbouring aerobic cells) in some, but not all mouse tumours (Foster *et al.*, 1976;

Brown, 1977; Denekamp, 1978; Brown & Yu, 1979).

The preceding paper (Tannock, 1980) reported a study of the interaction in mice of methotrexate, 5-fluorouracil or Adriamycin with MISO or METRO, using the endpoints of growth delay of two experimental tumours, and of weight loss and myelosuppression to assess host toxicity. I now report similar studies of the combination of CY with single or multiple doses of MISO or METRO and of BCNU with single-dose MISO. I include experiments designed to study the mechanisms underlying the large interactions that have been observed.

### MATERIALS AND METHODS

C3H male mice were used in all experiments, and the experimental tumours were the KHT fibrosarcoma and the 16/C mammary adenocarcinoma (Lin & Bruce, 1972; Corbett *et al.*, 1978). Characteristics of these tumours, techniques for implantation and assessment of tumour growth, and methods for measuring haemoglobin level (Hb) and white blood cell counts (WBC) on peripheral blood, have been described in the preceding paper (Tannock, 1980). Unless stated otherwise, mice received drug treatment when their tumours were 0·3–0·5 g in size. All measurements were made without knowledge of the treatment history of the animals, and most experiments were repeated to check reproducibility.

For treatment of the mice, Cyclophosphamide (Horner), Misonidazole (Roche), and Metronidazole (Poulenc) were dissolved in saline for injection, while BCNU was dissolved in 10% ethanol. Drugs were given by i.p. injection in a fluid volume of 0·1 mg/g body weight (CY or BCNU) or 0·02–0·05 ml/g (MISO or METRO). Single injections of CY or BCNU were used in all experiments. MISO was injected usually as a single dose of 1 mg/g body weight, but multiple i.p. injections of MISO or METRO were given in some experiments in combination with CY. Nine doses of 0·2 mg/g/injection (MISO) or 0·4 mg/g/injection (METRO) were injected at 4h intervals in an attempt to sustain serum levels over 36 h (Tannock, 1980).

The mechanism of interaction of MISO with CY or BCNU was investigated by exposing Chinese hamster ovary (CHO) cells to serum from mice that had been treated with drugs. Blood was obtained from the inferior vena cava of mice that had received CY or BCNU alone, MISO alone, or a combination. Heparin was injected shortly before death to prevent clotting, and the interval between drug treatment and death was 0·5 or 1·0 h. Pooled blood from several mice was placed on ice and centrifuged; the serum was filtered, and 0·5 ml volumes of serum from mice that had received different treatments were added to vials containing 7·5 ml of a suspension of CHO cells. The cells were in complete α-medium supplemented with antibiotics and 10% foetal calf serum (FCS) at a concentration of $5 \times 10^5$/ml and were agitated gently with a magnetic stirring bar. The cells were exposed to the murine serum at 37°C for periods of up to 6 h under either aerobic or hypoxic conditions. A humidified gas mixture of either 95% air/5% $CO_2$ or 95% $N_2$/5% $CO_2$ (<10 pts/$10^6$ $O_2$) flowed through inlet and outlet tubes in the stoppers of each vial from 1 h before adding serum until the end of the 6h exposure (Mohindra & Rauth, 1976). At various times cells were withdrawn from the vials with a 100μl pipette, centrifuged and resuspended in fresh medium. Appropriate dilutions were plated in triplicate Petri dishes. Estimates of cell survival were obtained by counting stained colonies about 9 days later.

### RESULTS

### *Misonidazole or Metronidazole alone*

Data presented in the preceding paper (Tannock, 1980) show that single-dose MISO (1 mg/g), and multiple injections of either MISO or METRO have no effect on the growth of either the KHT or 16/C tumours. Single-dose MISO given after 15 Gy radiation to the 16/C tumour led to a small increase in delay to regrowth, implying some ability to kill hypoxic cells spared by radiation; in contrast, there was no increase in growth delay compared to irradiation alone of the KHT sarcoma when single-dose MISO (1 mg/g) or multiple doses of either drug were given after radiation. MISO and METRO in the dose and schedule used were well tolerated, and usually produced only transient weight

FIG. 1.—Growth curves for the KHT sarcoma in mice treated with saline (○), cyclophosphamide (75 mg/kg) (●) or CY+MISO (1 mg/g) (△). The drugs were given simultaneously (A), or MISO was given 4 h before CY (B). Means ± s.e. are plotted for 7 mice.

loss, < 5%. Peripheral WBC counts after MISO were within the normal range, but means were slightly below control animals.

*Cyclophosphamide*

(i) *Anti-tumour effects.*—Both the KHT and 16/C tumours responded to CY, and at higher doses complete regressions and growth delay of two weeks or more were obtained. The combination of MISO (1 mg/g) with CY led to increased delay to regrowth of both tumours (Figs 1–3, Table I). This effect was present when MISO was injected up to 4 h before CY and when the drugs were given simultaneously (Figs 1 & 2). Further experiments with the KHT tumour showed that the increased anti-tumour effect of the drug combination was lost if MISO was given 24 h before, or 2 h after CY.

Delay to regrowth of the KHT tumour was also increased by combining CY with the multiple dose schedule of MISO or METRO (Table I, Fig. 3c); in these experiments CY (50 or 75 mg/kg) was injected with the 5th of 9 doses of the nitroimidazole. Despite the longer exposure of tumour cells to MISO or METRO, this schedule was no more effective for increasing the anti-tumour effect of CY than a single dose of MISO.

In one experiment, CY was combined with a course of multiple injections of Ro-05-9963 (0·4 mg/kg × 9), the O-demethylation product of MISO. There was no significant increase in the anti-tumour effect of CY or of host toxicity.

Further experiments were designed to test whether MISO or METRO were acting in part to kill hypoxic cells that

FIG. 2.—Growth curves for the 16/C carcinoma in mice treated with saline (○), CY (100 mg/kg in A, 75 mg/kg in B) (△), MISO (1 mg/g) (●) or simultaneous CY+MISO (▲). In (B) MISO was also given 1 h before CY (▼). Means±s.e. for groups of 6–8 mice are indicated.

were spared by CY. These experiments compared treatment of the KHT tumour with CY alone or combined with MISO or METRO under the following conditions:

(a) Treatment of small tumours that are known to contain fewer hypoxic cells (Hill, 1980).
(b) Use of lower doses of MISO that are known to be ineffective for killing hypoxic cells.
(c) Treatment of animals that were kept warm, because hypoxic-cell toxicity of nitroimidazoles depends on temperature, and peripheral tumours in the leg might be cooler than core body temperature (Stratford & Adams, 1978).

The results of some of these experiments are shown in Fig. 3, and do not support a role for MISO or METRO in killing hypoxic KHT cells. Treatment of tumours on Day 5 after implantation (before they were palpable) led to an equal or greater increase in growth delay for the drug combination than treatment of larger tumours (Fig. 3A). The increase in growth delay after adding MISO to CY for treatment of 0·3–0·5 g tumours depended on dose of MISO, but was detectable at a dose of 0·5 mg/g body weight (Fig. 3B). Maintaining animals in an incubator at 37°C for 4 h after treatment with MISO (1 mg/g) led to death of the animals: the warm environment presumably prevented the known effect of MISO in causing transient decrease in body temperature, and confirms a previous report of the toxicity of keeping animals warm after MISO (Gomer & Johnson, 1979). Mice tolerated an environment of 35°C for 4 h after single-dose MISO, or for 36 h during a course of multiple injections, but it is not known whether the ambient temperature of 35°C led to an increase in tumour temperature. There was no con-

FIG. 3.—Growth curves for the KHT sarcoma treated with saline (○), CY (●) or CY+MISO (△) or (CY+METRO) (▽). Experimental conditions were as follows: (A) CY (75 mg/kg) and MISO (1 mg/g) given to mice bearing non-palpable tumours on Day 5 after transplantation; (B) CY (75 mg/kg) and MISO (0·5 mg/g) given to mice bearing 0·4–0·5g tumours; (C) CY (75 mg/kg) and METRO (0·4 mg/g x 9) given to mice bearing 0·3g tumours. One group of mice (▼) was kept at 35°C throughout the 36h course of injections. CY was given simultaneously with MISO, or with the 5th of 9 doses of METRO. Means ± s.e. are plotted for 6–8 mice.

sistent change to delay in tumour regrowth for mice kept warm during and after treatment with CY alone, MISO or METRO alone, or the combination, when compared to experiments at room temperature (*e.g.* Fig. 3C).

(ii) *Host toxicity*.—The addition of MISO or METRO to treatment with CY led to more deaths and more weight loss (Table I). The increase in host toxicity was greater for those conditions that led to the larger increases in anti-tumour effects. In an attempt to study the effect of adding MISO to CY on Therapeutic Index, growth delay for the KHT sarcoma and host toxicity were compared for a large dose of CY, and a smaller dose combined with MISO. CY at a dose of 200 mg/kg gave a mean growth delay of 17·5 days, whereas CY (75 mg/kg)+MISO (1 mg/g) gave similar or greater toxicity, but caused mean growth delay of only 10 days (Table I). Therapeutic Index is thus lower for combined treatment.

The addition of MISO to CY also led to greater myelosuppression. The serial removal of small blood samples from the tail veins of mice led to a fall in Hb and a compensatory increase in WBC count, and these effects were greater in mice with tumours. MISO alone had no significant effect on the Hb or WBC count, whereas CY (75 mg/kg) caused a reduction in WBC to a nadir at 3–4 days after treatment, followed by rapid recovery. The addition of MISO (1 mg/g) led to a sig-

TABLE 1.—*Treatment-related death, weight loss and mean growth delay of the KHT sarcoma after treatment with CY alone, or in combination with MISO or METRO*

| | Mean tumour growth delay* (days) (range†) | % Weight loss (range†) | Proportion of deaths from treatment‡ |
|---|---|---|---|
| CY (75 mg/kg) alone | 6 (5–7) | 7 (3–12) | 1/53 |
| CY (75 mg/kg)+MISO (0·5 mg/g) | 8·5 | 13 | 0/8 |
| CY (75 mg/kg)+MISO (1 mg/g) | 10 (8–12) | 16 (10–21) | 13/64 |
| CY (75 mg/kg)+MISO (0·2 mg/g × 9) | 8 (7–8·5) | 12 (10–15) | 1/15 |
| CY (75 mg/kg)+METRO (0·4 mg/g × 9) | 9 (7·5–10) | 21 (19–23) | 3/15 |
| CY (200 mg/kg) alone | 17·5 (17–18) | 13 (10–16) | 2/15 |

* Displacement between tumour growth curves for treated and control animals at a tumour size of 1 g.
† Range of mean values from individual experiments.
‡ Deaths occurred 7–10 days after treatment. Deaths due to the tumour were not observed at this time.

FIG. 4.—Total WBC count measured serially on blood samples from tumour-bearing C3H mice. Mice were treated with saline (○), MISO (1 mg/g) (△), CY (75 mg/kg) (●), or a combination of the drugs given simultaneously (▲). Means ± s.e. for 7–8 samples are indicated.

nificant decrease in the nadir of WBC count (Fig. 4). In several other experiments the WBC and absolute polymorph count were measured from blood smears on the 3rd or 4th day after treatment with CY, alone or in combination with single or multiple doses of MISO or METRO. In all experiments the blood counts were lower after combined treatment.

(iii) *Mechanism.*—Serum from mice given CY was active against CHO cells *in vitro*, and this system provides a bioassay for active metabolites of the drug. The level of survival of CHO cells varied slightly between experiments, and the activity of the metabolites of CY decayed even when serum was frozen immediately after preparation. Thus, serum was prepared as rapidly as possible before adding to cultures of CHO cells in order to minimize variability in time of preparation.

Serum from untreated mice, and from mice that had received MISO (1 mg/g) 0·5 or 1·0 h earlier, had no effect on aerobic or hypoxic CHO cells. This result is expected because the 1:16 dilution of the serum in culture led to levels of MISO much lower than those usually lethal to hypoxic cells. Serum from mice given CY (200 mg/kg) 0·5 h earlier was active, but most of this activity was lost 1 h after treatment (Fig.5). Serum from mice that had received CY and MISO had similar activity to serum from mice receiving CY alone at 0·5 h, but retained much greater activity at 1·0 h (Fig. 5). There was little difference in response of aerobic and hypoxic cells. Thus MISO seems to delay the excretion or inhibit the breakdown of active metabolites of CY.

### *BCNU*

*Anti-tumour effects and host toxicity.*—BCNU caused tumour regression and

FIG. 5.—Survival of aerobic (open symbols) or hypoxic (closed symbols) CHO cells treated with serum from mice that had received MISO 1 mg/g (▽ or ▼), CY 200 mg/kg (○ or ●) or both drugs (△ or ▲). (A) Data from 2 experiments for serum removed 0·5 h after treatment. (B) Data from 1 experiment for serum removed 1·0 h after treatment. (A repeat experiment gave qualitatively similar results but a different level of survival after combined treatment.) Mean survival and range of estimates from triplicate Petri dishes are plotted.

delayed growth of the KHT sarcoma (Lin & Bruce, 1972) but experiments in this and other laboratories have shown the drug to be inactive against the 16/C carcinoma (Corbett *et al.*, 1978). Combination of BCNU with single-dose MISO led to a marked increase in effectiveness against the KHT tumour (Fig. 6 and Table II).

The nature of the anti-tumour interaction was investigated in experiments analagous to those performed for CY. The anti-tumour effects of BCNU and MISO were observed when the drugs were given together but not when MISO preceded BCNU by 4 h. MISO led to slightly greater growth delay when combined with BCNU for treatment of small non-palpable tumours than for treatment of larger tumours (Fig. 6). The increased effect of the combination was dependent on the dose of MISO, but was seen at doses of 0·5 or 0·75 mg/g (*e.g.* Fig. 6c).

The combination of BCNU and MISO caused more deaths and more weight loss than the same dose of BCNU alone (Table II). For comparison of Therapeutic Index, the anti-tumour effects and toxicity of a large dose of BCNU alone were compared with those of a smaller dose of BCNU with MISO. The data of Table II suggest about equal toxicity for the same delay in tumour growth and hence no effect on Therapeutic Index.

Treatment with BCNU had no effect on Hb level, and caused only a small decrease in WBC count. The addition of MISO had

FIG. 6.—Growth curves for the KHT sarcoma treated with saline (○), BCNU (●), or simultaneous BCNU+MISO (△). Experimental conditions were as follows: (A) BCNU (20 mg/kg) and MISO (1 mg/g) given to mice bearing ≈ 0·3g tumours; (B) Same doses to mice bearing non-palpable tumours on Day 5 after transplantation; (C) BCNU (33 mg/kg) and MISO (0·75 mg/g) given to mice bearing 0·4–0·5g tumours. Means ± s.e. for groups of 6–8 mice are plotted.

little effect on the nadir of WBC count after treatment (Table III).

(ii) *Mechanism.*—Serum from mice given BCNU (66 mg/kg) 0·5 h earlier was active against CHO cells *in vitro*, and there was no difference in the response of aerobic or hypoxic CHO cells (Fig. 7). Serum from mice treated 1 h earlier was inactive. Serum from animals receiving combined treatment 0·5 h before death (BCNU 66 mg/kg +MISO 1 mg/g) had similar activity for aerobic cells to that from mice receiving BCNU alone, but was much more toxic for hypoxic cells (Fig. 7). This unexpected result has been confirmed in repeated experiments. Thus MISO, at a dose that is ineffective for killing hypoxic cells, seems capable of selectively "sensitizing" hypoxic cells to the action of BCNU.

The role of the above effect in causing the increased anti-tumour effects *in vivo* for the combination of BCNU and MISO

TABLE II.—*Treatment-related death, weight loss, and mean growth delay of the KHT sarcoma after treatment with BCNU alone, or in combination with MISO*

| | Mean tumour growth delay (days) (range) | % Weight loss (range) | Proportion of deaths from treatment* |
|---|---|---|---|
| BCNU (20 mg/kg) alone | 1·5 (1·5–2·0) | 2 (0–6) | 0/29 |
| BCNU (20 mg/kg)+MISO (0·5 mg/g) | 3·5 | 7 | 0/8 |
| BCNU (20 mg/kg)+MISO (1 mg/g) | 8·5 (7·5–10) | 15 (11–19) | 1/30 |
| BCNU (33 mg/kg) alone | 5·0 | 9 | 0/8 |
| BCNU (33 mg/kg)+MISO (0·75 mg/g) | 16·0 | 27 | 3/8 |
| BCNU (50 mg/kg) alone | 14·0 | 23 | 2/8 |

* Deaths within the first 2 weeks after treatment following progressive weight loss.

TABLE III.—*Total white blood cell count* ($\times 10^3$) *at various times after treatment with BCNU* (33 *mg/kg*) *alone, or in combination with MISO* (1 *mg/g*)

| | Day 2 | Day 3 | Day 4 | Day 6 |
|---|---|---|---|---|
| Controls | 10·4±0·6 | 12·3±0·5 | 12·6±0·5 | 12·4±0·8 |
| BCNU | 7·9±0·3 | 7·4±0·5 | 5·9±0·4 | 10·1±0·6 |
| BCNU+MISO | 9·9±0·6 | 5·6±0·5 | 5·4±0·5 | 10·9±0·5 |

FIG. 7.—Survival of aerobic (open symbols) or hypoxic (closed symbols) CHO cells treated with serum from mice that had been treated with BCNU (66 mg/kg) with 1 mg/g MISO (triangles) or without (circles). Mean and range from triplicate Petri dishes are plotted.

cannot be determined. However, the increase in host toxicity cannot easily be explained by specific effects on hypoxic cells, and other undetermined drug interactions must be presumed.

### DISCUSSION

I have shown that MISO or METRO may influence the activity of CY or BCNU when the drugs are injected into mice. The drug interactions lead to increased responses of experimental tumours, but also to increased toxicity. Combined treatment does not lead to therapeutic advantage, and may be detrimental.

The interaction of CY and MISO seems to be due in part to a change in the pharmacokinetics of CY. Results presented in Fig. 5, and confirmed in other experiments, have shown that the administration of MISO with CY leads to a longer retention in serum of metabolites toxic for mammalian cells. This mechanism probably causes the increased growth delay found in both experimental tumours, and the increase in host toxicity, including myelosuppression. The cause of the decrease in Therapeutic Index for the combination of MISO and a moderate dose of CY, as compared to a higher dose of CY alone, is undetermined. The change in pharmacokinetics might lead to selective retention of those metabolites of CY that have a high ratio of host toxicity to antitumour effects, or there may be other independent drug reactions that lead to an increase in deaths and weight loss.

The interaction of BCNU and MISO is complex. The combination has been shown to cause an increase in non-specific host toxicity, and the nature of this interaction is unknown. However, MISO, at a concentration ineffective for killing hypoxic cells, has been shown to give a selective "sensitization" for the action of BCNU against hypoxic cells. There was a similar effect when BCNU and MISO were added directly to cultures of CHO cells. Experiments are being performed in tissue culture to characterize this interaction, and will be reported separately. BCNU was found to spare hypoxic cells in one experimental tumour (Hill & Stanley, 1975) but it remains uncertain whether the observed interaction of BCNU and MISO for hypoxic cells is a cause of the increased activity of the combination for the KHT tumour.

The present experiments, and those of the previous paper (Tannock, 1980), were undertaken to seek evidence for killing by nitroimidazoles of hypoxic cells which might have been spared by conventional chemotherapy, and to provide data on drug interactions prior to the use of such combinations in man. There is little evidence from the present experiments for selective killing of hypoxic cells in murine tumours by MISO or METRO given with anti-cancer drugs, since anti-tumour effects have been accompanied by equal or even greater host toxicity. Little is known about the relative response of aerobic and hypoxic clonogenic tumour cells to chemotherapy. Such information is important, for if surviving cells were predominantly hypoxic, nitroimidazoles and other drugs should be selected and developed for their hypoxic cell toxicity as well as for hypoxic-cell radiosensitization. The added possibility of hypoxic-cell "chemosensitization", suggested by the above results for BCNU and MISO is another potentially exploitable mechanism, despite the lack of therapeutic advantage for the KHT sarcoma.

A major conclusion of the present studies is that MISO or METRO lead to a marked increase in host toxicity when combined with several anti-cancer drugs. Combination of misonidazole or metronidazole with cyclophosphamide or BCNU in patients should be undertaken with great caution, and should follow the design of a Phase I trial.

I wish to thank Mrs P. Guttman for her expert assistance and Dr R. P. Hill for constructive criticism. Supported by a Reaserch Grant from the National Cancer Institute of Canada.

### REFERENCES

Brown, J. M. (1977) Cytotoxic effects of the hypoxic cell radiosensitizer Ro-07-0582 to tumor cells *in vivo*. *Radiat. Res.*, **72**, 469.

Brown, J. M. & Yu, N. Y. (1979) Cytotoxicity of misonidazole *in vivo* under conditions of prolonged contact of drug with the tumour cells. *Br. J. Radiol.*, **52**, 893.

Corbett, T. H., Griswold, D. P., Jr, Roberts, B. J., Peckham, J. C. & Schabel, F. M., Jr (1978) Biology and therapeutic response of a mouse mammary adenocarcinoma (16/C) and its potential as a model for surgical adjuvant chemotherapy. *Cancer Treat. Rep.*, **62**, 1471.

Denekamp, J. (1978) Cytotoxicity and radiosensitization in mouse and man. *Br. J. Radiol.*, **51**, 636.

Dixon, B., Moore, J. V. & Speakman, H. (1978) Radiobiological hypoxia of a transplanted rat tumour and the effect of treatment with cyclophosphamide. *Eur. J. Cancer*, **14**, 1383.

Foster, J. L., Conroy, P. J., Searle, A. J. & Willson, R. L. (1976) Metronidazole (Flagyl): Characterization as a cytotoxic drug specific for hypoxic tumour cells. *Br. J. Cancer*, **33**, 485.

Gomer, C. J. & Johnson, R. J. (1979) Relationship between misonidazole toxicity and core temperature in C3H mice. *Radiat. Res.*, **78**, 329.

Hill, R. P. (1980) An appraisal of *in vivo* assays of excised tumours. *Br. J. Cancer*, **41**, Suppl. IV, p. 230.

Hill, R. P. & Stanley, J. A. (1975) The response of hypoxic B16 melanoma cells to *in vivo* treatment with chemotherapeutic agents. *Cancer Res.*, **35**, 1147.

Hirst, D. G. & Denekamp, J. (1979) Tumour cell proliferation in relation to the vasculature. *Cell Tissue Kinet.*, **12**, 31.

Lin, H. & Bruce, W. R. (1972) Chemotherapy of the transplanted KHT fibrosarcoma in mice. *Ser. Haematol.*, **5**, 89.

Mohindra, J. K. & Rauth, A. M. (1976) Increased cell killing by metronidazole and nitrofurazone of hypoxic compared to aerobic mammalian cells. *Cancer Res.*, **36**, 930.

Sridhar, R., Koch, C. & Sutherland, R. M. (1976) Cytotoxicity of two nitroimidazole radiosensitizers in an *in vitro* tumour model. *Int. J. Radiat. Oncol. Biol. Phys.*, **1**, 1149.

Stratford, I. J. & Adams, G. E. (1977) Effect of hyperthermia on differential cytotoxicity of a hypoxic cell radiosensitizer, Ro-07-0582, on mammalian cells *in vitro*. *Br. J. Cancer*, **35**, 307.

Stratford, I. J. & Adams, G. E. (1978) The toxicity of the radiosensitizer misonidazole towards hypoxic cells *in vitro*: A model for mouse and man. *Br. J. Radiol.*, **51**, 745.

Tannock, I. F. (1968) The relation between cell proliferation and the vascular system in a transplanted mouse mammary tumour. *Br. J. Cancer*, **22**, 258.

Tannock, I. F. (1970) Population kinetics of carcinoma cells, capillary endothelial cells, and fibroblasts in a transplanted mouse mammary tumour. *Cancer Res.*, **30**, 2470.

Tannock, I. F. (1980) The *in vivo* interaction of anti-cancer drugs with misonidazole or metronidazole: Methotrexate, 5-fluorouracil and adriamycin. *Br. J. Cancer*, **42**, 861.

Taylor, Y. C. & Rauth, A. M. (1978) Differences in the toxicity and metabolism of the 2-nitroimidazole misonidazole (Ro-07-0582) in HeLa and Chinese hamster ovary cells. *Cancer Res.*, **38**, 2745.


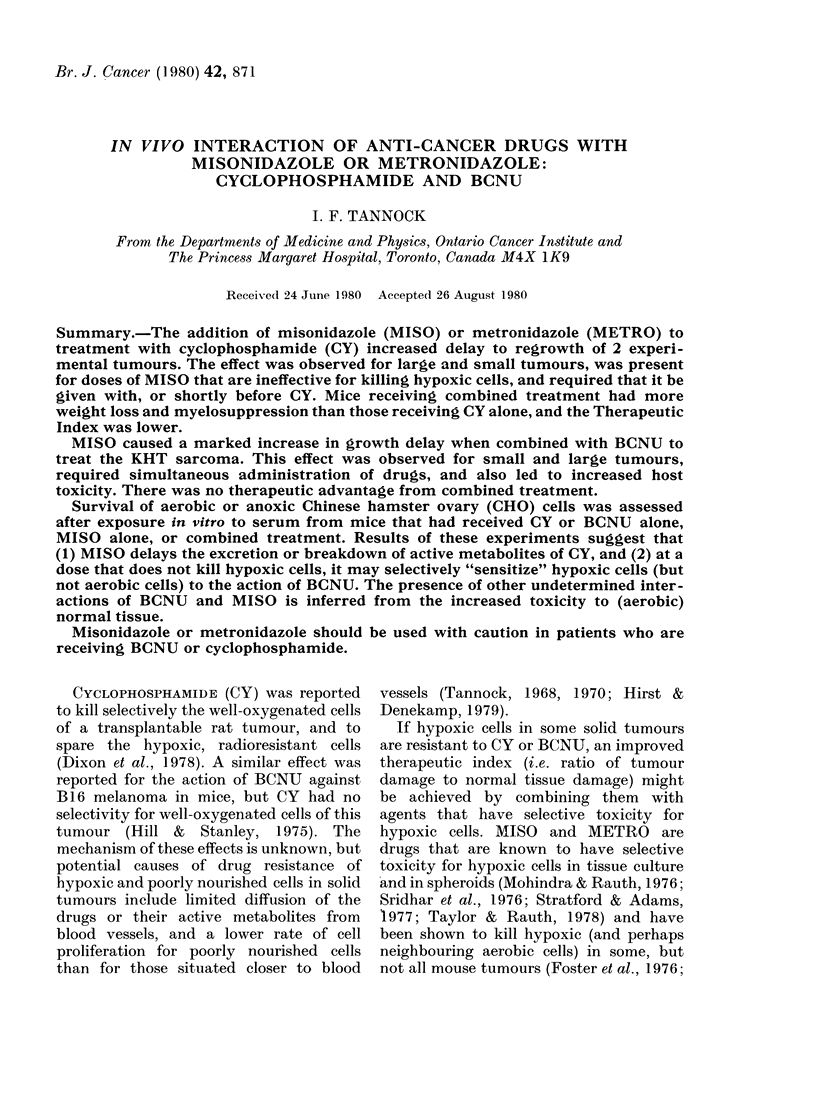

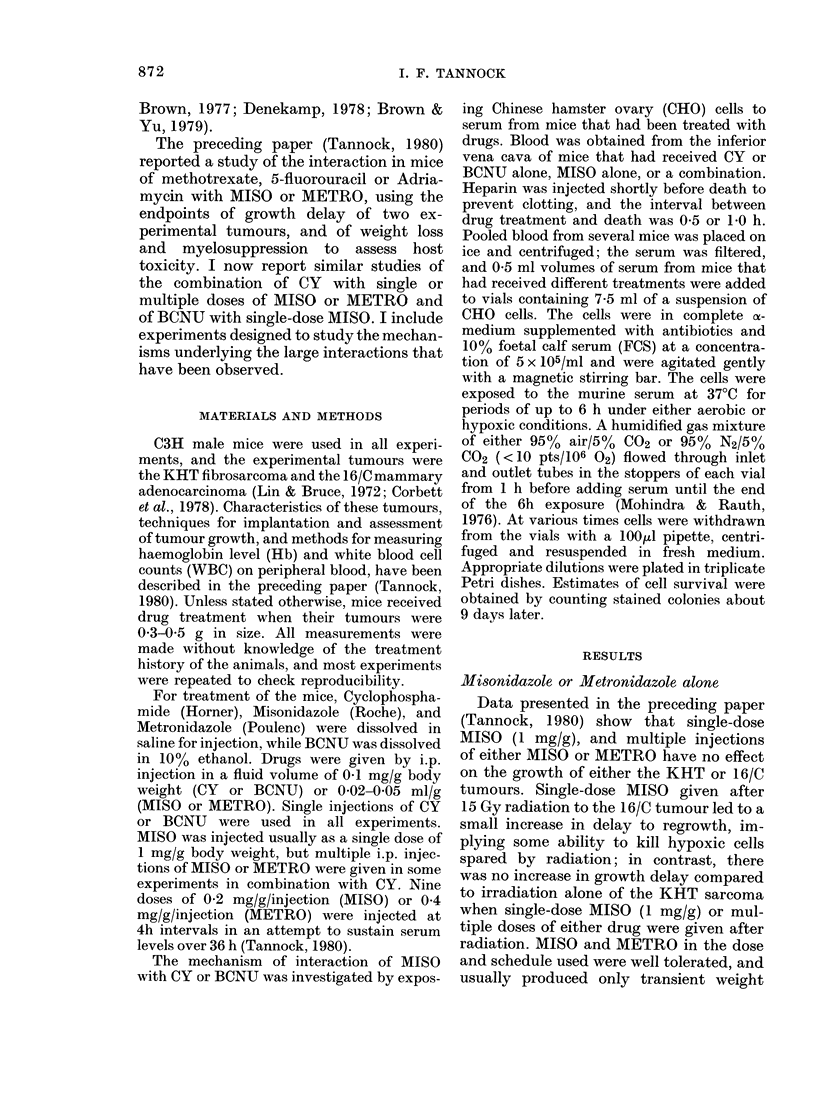

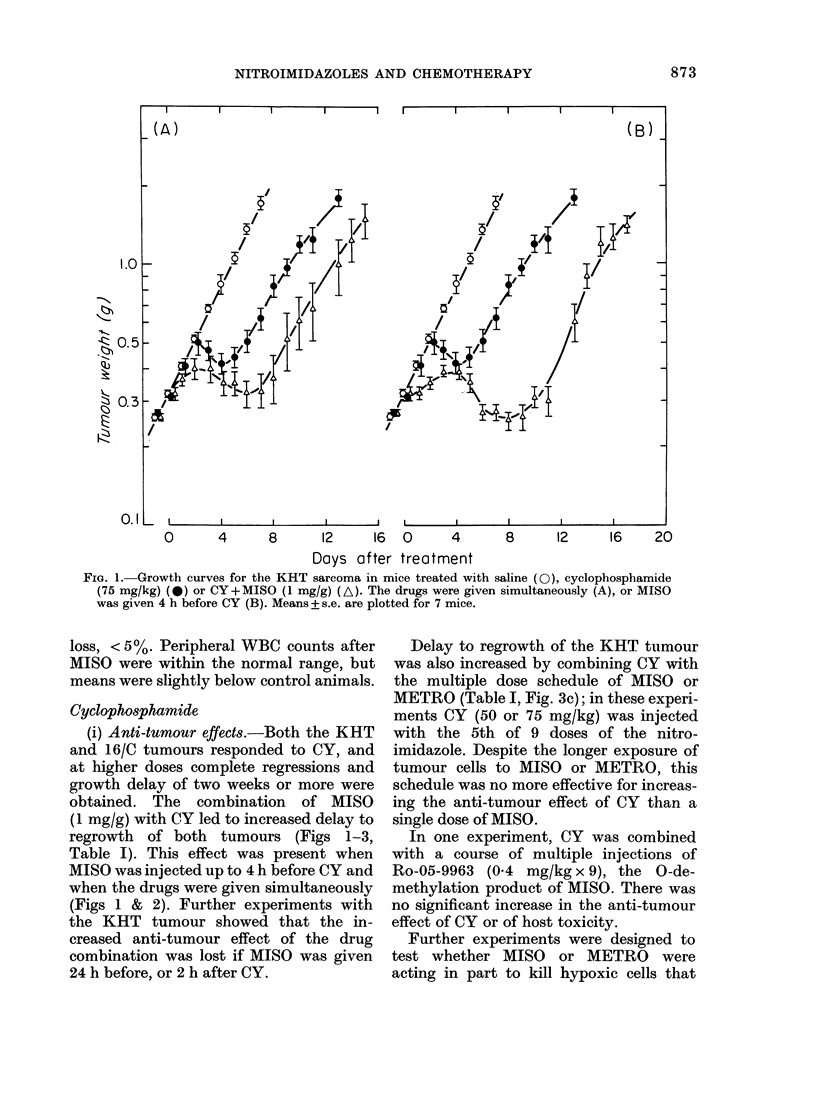

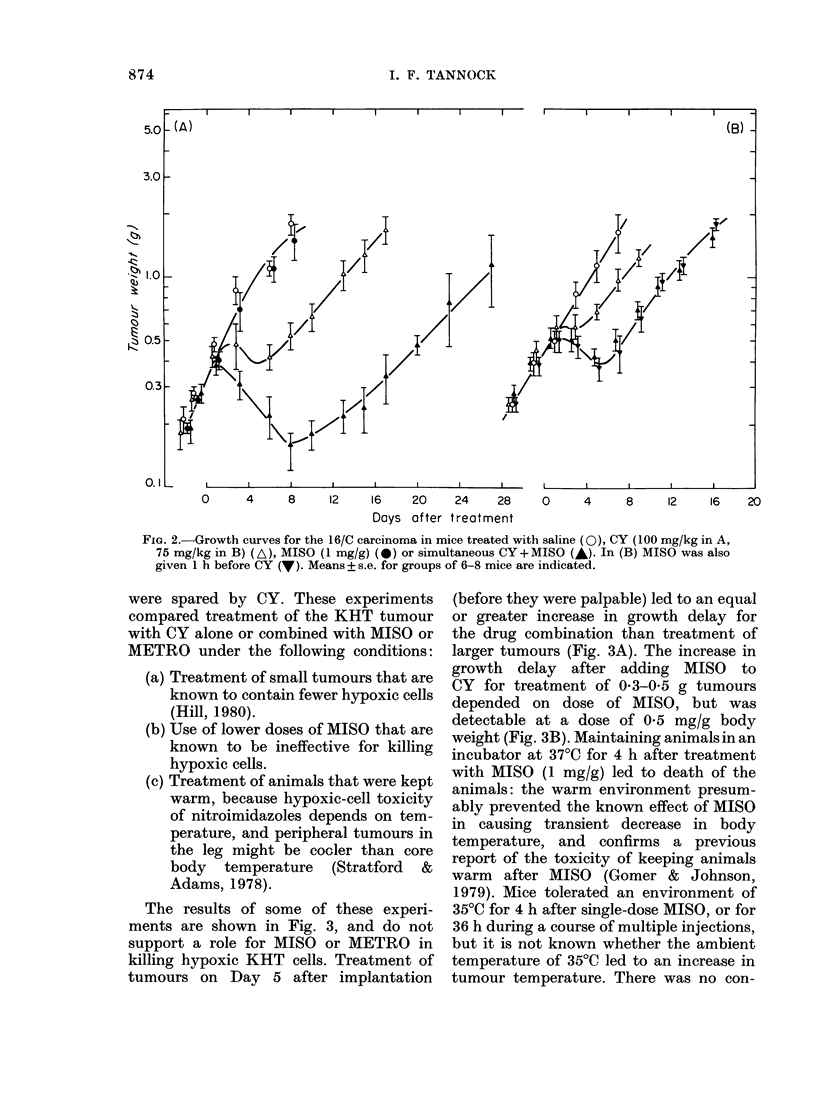

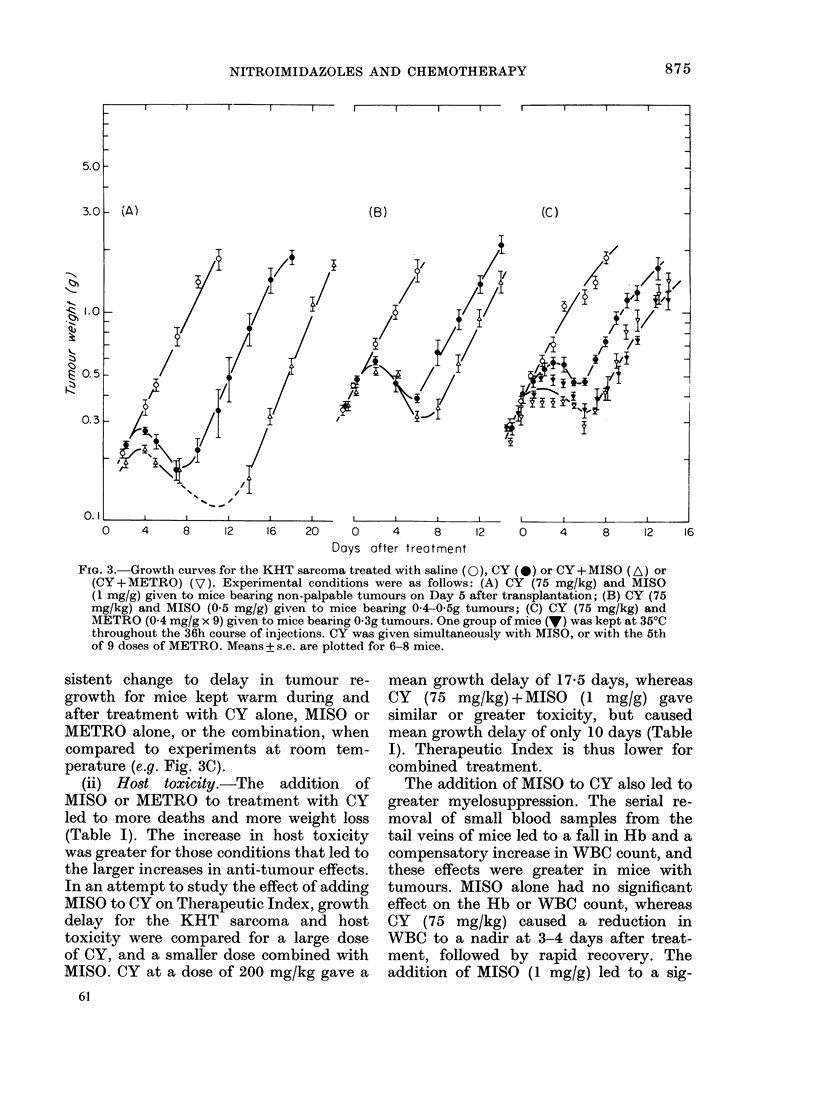

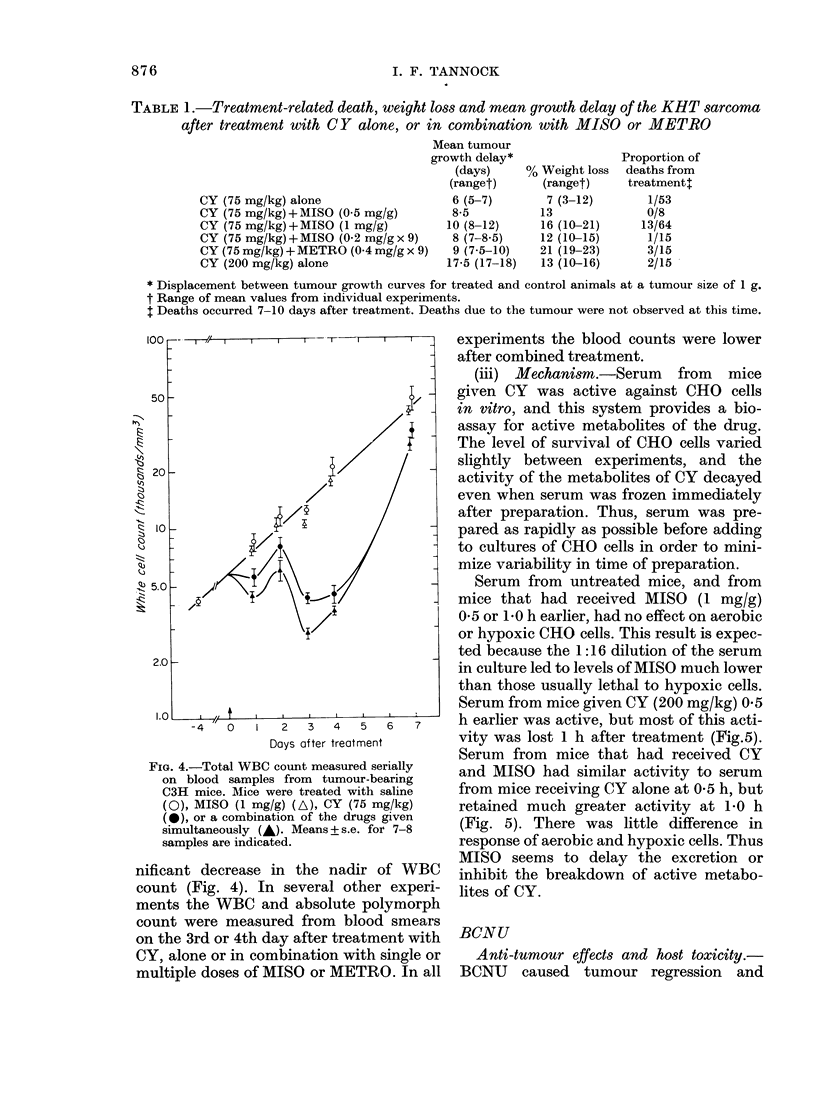

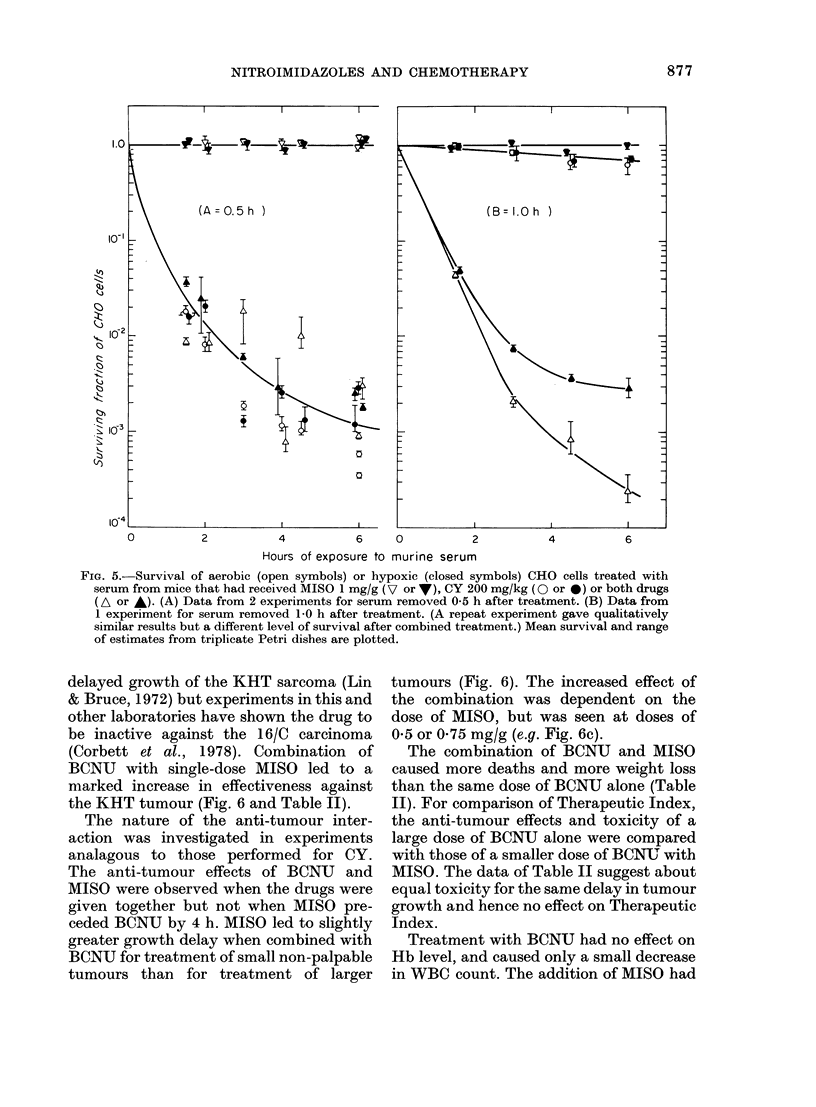

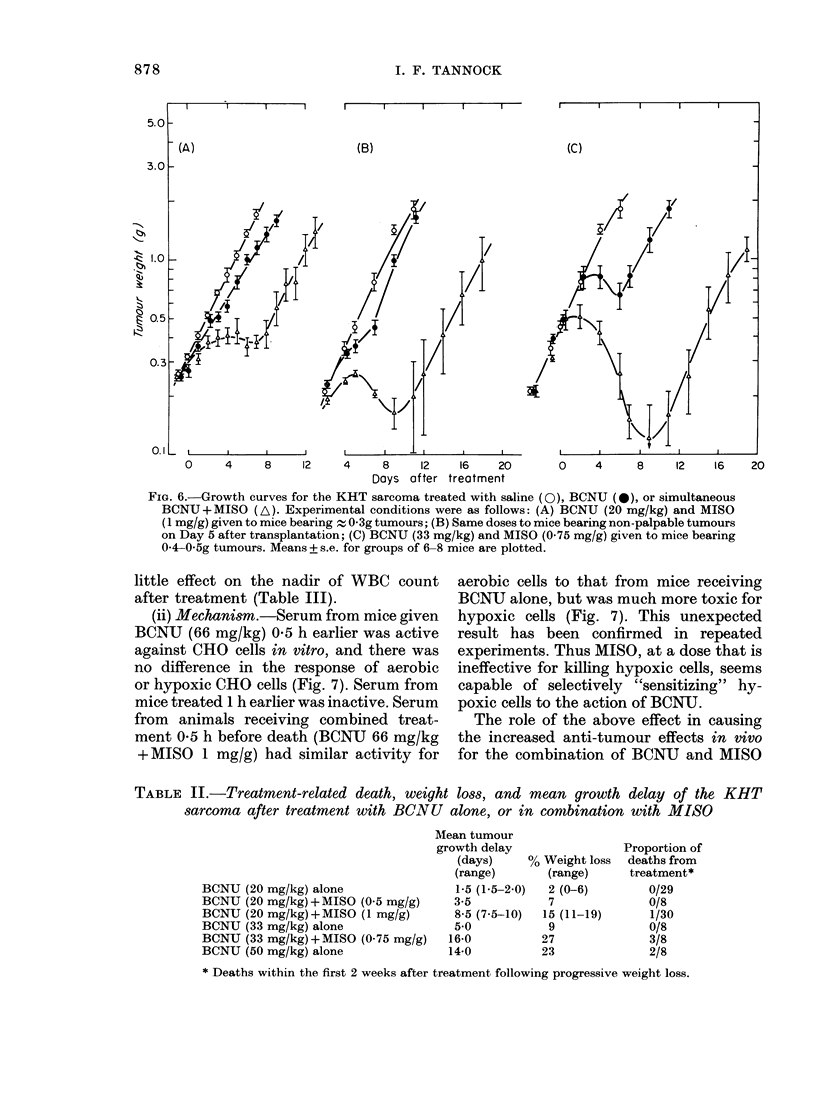

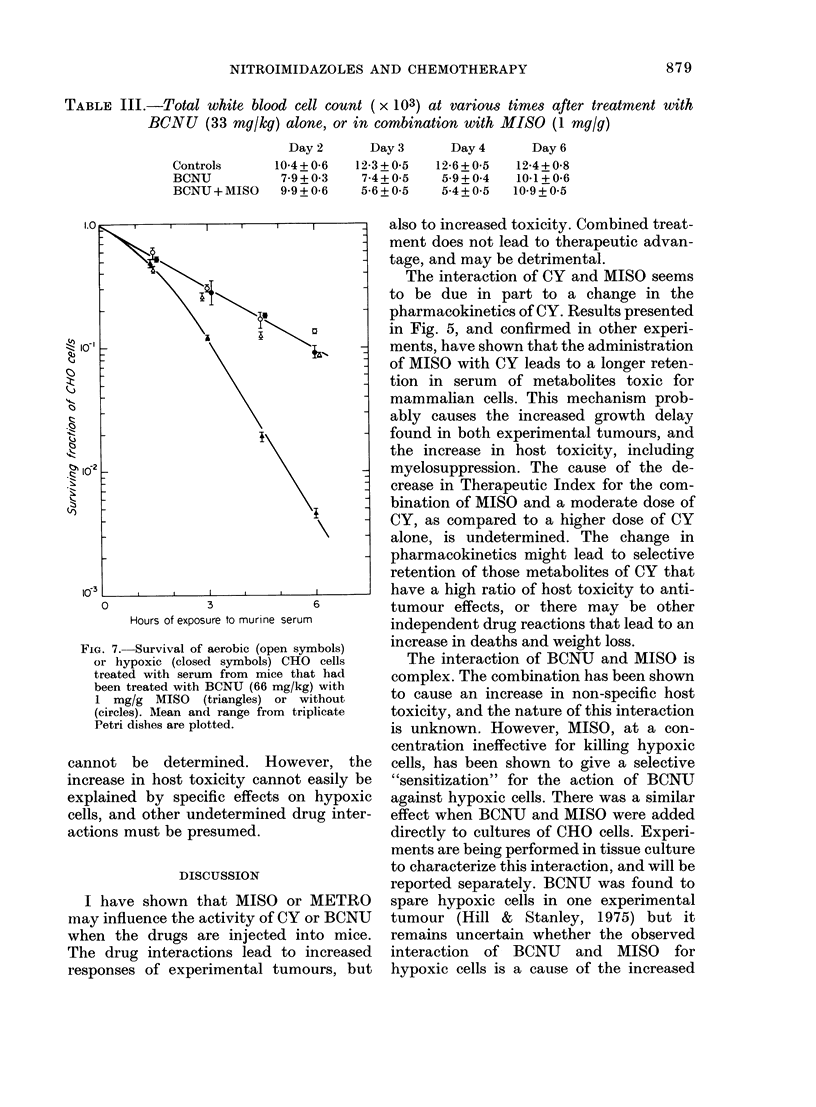

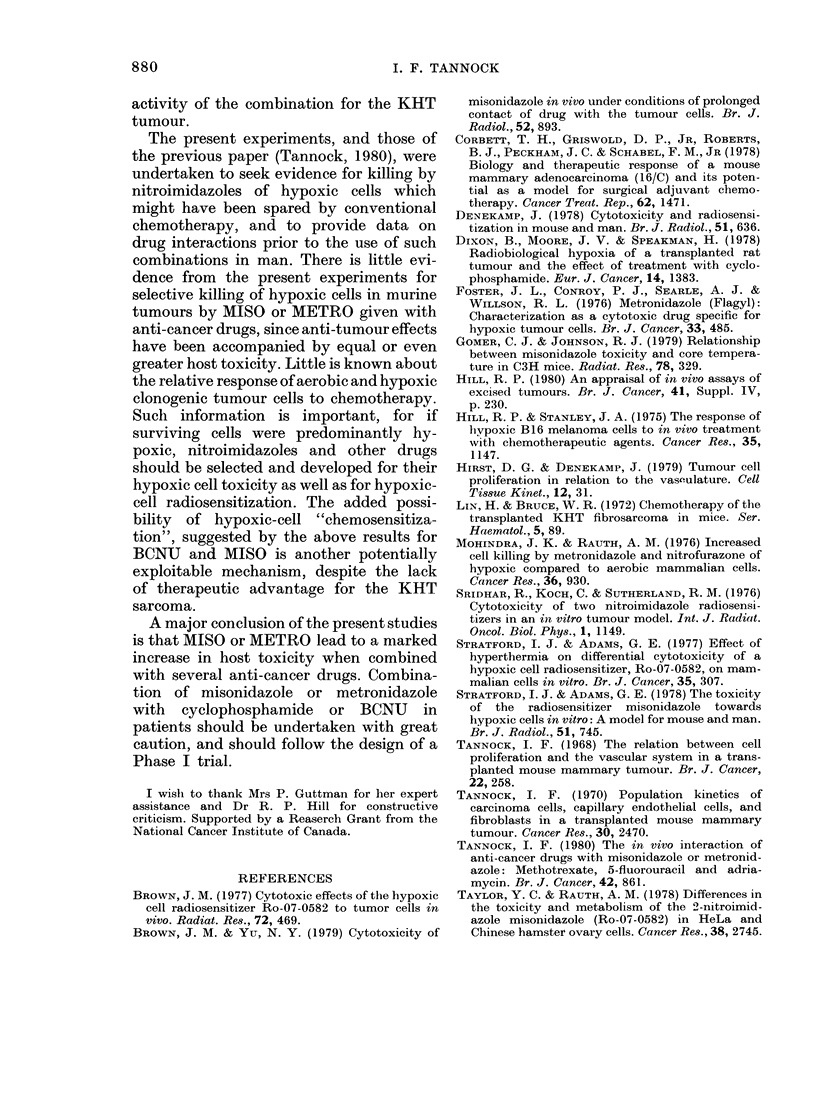


## References

[OCR_00908] Brown J. M. (1977). Cytotoxic effects of the hypoxic cell radiosensitizer Ro 7-0582 to tumor cells in vivo.. Radiat Res.

[OCR_00913] Brown J. M., Yu N. Y. (1979). Cytotoxicity of misonidazole in vivo under conditions of prolonged contact of drug with the tumour cells.. Br J Radiol.

[OCR_00919] Corbett T. H., Griswold D. P., Roberts B. J., Peckham J. C., Schabel F. M. (1978). Biology and therapeutic response of a mouse mammary adenocarcinoma (16/C) and its potential as a model for surgical adjuvant chemotherapy.. Cancer Treat Rep.

[OCR_00927] Denekamp J. (1978). Cytotoxicity and radiosensitization in mouse and man.. Br J Radiol.

[OCR_00930] Dixon B., Moore J. V., Speakman H. (1978). Radiobiological hypoxia of a transplanted rat tumour and the effect of treatment with cyclophosphamide.. Eur J Cancer.

[OCR_00936] Foster J. L., Conroy P. J., Searle A. J., Willson R. L. (1976). Metronidazole (Flagyl): characterization as a cytotoxic drug specific for hypoxic tumour cells.. Br J Cancer.

[OCR_00942] Gomer C. J., Johnson R. J. (1979). Relationship between misonidazole toxicity and core temperature in C3H mice.. Radiat Res.

[OCR_00947] Hill R. P. (1980). An appraisal of in vivo assays of excised tumours.. Br J Cancer Suppl.

[OCR_00952] Hill R. P., Stanley J. A. (1975). The response of hypoxic B16 melanoma cells to in vivo treatment with chemotherapeutic agents.. Cancer Res.

[OCR_00958] Hirst D. G., Denekamp J. (1979). Tumour cell proliferation in relation to the vasculature.. Cell Tissue Kinet.

[OCR_00963] Lin H., Bruce W. R. (1972). Chemotherapy of the transplanted KHT fibrosarcoma in mice.. Ser Haematol.

[OCR_00968] Mohindra J. K., Rauth A. M. (1976). Increased cell killing by metronidazole and nitrofurazone of hypoxic compared to aerobic mammalian cells.. Cancer Res.

[OCR_00974] Sridhar R., Koch C., Suterland R. (1976). Cytotoxicity of two nitroimidazole radiosensitizers in an in vitro tumor model.. Int J Radiat Oncol Biol Phys.

[OCR_00980] Stratford I. J., Adams G. E. (1977). Effect of hyperthermia on differential cytotoxicity of a hypoxic cell radiosensitizer, Ro-07-0582, on mammalian cells in vitro.. Br J Cancer.

[OCR_00986] Stratford I. J., Adams G. E. (1978). The toxicity of the radiosensitizer misonidazole towards hypoxic cells in vitro: a model for mouse and man.. Br J Radiol.

[OCR_01004] Tannock I. F. (1980). In vivo interaction of anti-cancer drugs with misonidazole or metronidazole: methotrexate, 5-fluorouracil and adriamycin.. Br J Cancer.

[OCR_00998] Tannock I. F. (1970). Population kinetics of carcinoma cells, capillary endothelial cells, and fibroblasts in a transplanted mouse mammary tumor.. Cancer Res.

[OCR_00992] Tannock I. F. (1968). The relation between cell proliferation and the vascular system in a transplanted mouse mammary tumour.. Br J Cancer.

[OCR_01010] Taylor Y. C., Rauth A. M. (1978). Differences in the toxicity and metabolism of the 2-nitroimidazole misonidazole (Ro-07-0582) in HeLa and Chinese hamster ovary cells.. Cancer Res.

